# Periodontitis and Alzheimer’s Disease: A Possible Comorbidity between Oral Chronic Inflammatory Condition and Neuroinflammation

**DOI:** 10.3389/fnagi.2017.00327

**Published:** 2017-10-10

**Authors:** Francisco B. Teixeira, Miki T. Saito, Filipe C. Matheus, Rui D. Prediger, Elizabeth S. Yamada, Cristiane S. F. Maia, Rafael R. Lima

**Affiliations:** ^1^Institute of Biological Science, Federal University of Pará, Belém, Brazil; ^2^College of Medicine, Federal University of Pará, Altamira, Brazil; ^3^Dental School, Brazil-Amazon Integrated Faculty, Belém, Brazil; ^4^Department of Pharmacology, Center of Biological Sciences, Federal University of Santa Catarina, Florianópolis, Brazil; ^5^Institute of Health Science, Federal University of Pará, Belém, Brazil

**Keywords:** Alzheimer disease, amyloid beta-peptides, dementia, inflammation, neurodegenerative diseases, neurofibrillary tangles, periodontal diseases, periodontitis

## Abstract

Periodontitis is an oral chronic infection/inflammatory condition, identified as a source of mediators of inflammation into the blood circulation, which may contribute to exacerbate several diseases. There is increasing evidence that inflammation plays a key role in the pathophysiology of Alzheimer’s disease (AD). Although inflammation is present in both diseases, the exact mechanisms and crosslinks between periodontitis and AD are poorly understood. Therefore, this article aims to review possible comorbidity between periodontitis and AD. Here, the authors discuss the inflammatory aspects of periodontitis, how this oral condition produces a systemic inflammation and, finally, the contribution of this systemic inflammation for worsening neuroinflammation in the progression of AD.

## Introduction

Life expectancy has increased considerably over the last three decades and the number of cases of dementia has expanded within aging population (Norton et al., 2014). Recent epidemiological projections indicate that the number of patients with dementia will more than triplicate by 2050 in comparison to 2010, and most of the cases of dementia are associated to Alzheimer’s disease (AD; Barnes and Yaffe, 2011; Norton et al., 2013, 2014).

Despite great efforts of researchers and clinicians, no effective disease-modifying drug has been approved for AD treatment to date (Norton et al., 2014; Bateman, 2015). Therefore, there is an increasing interest in identifying modifiable risk factors for dementia and AD, aiming to develop preventive strategies that could lower dementia prevalence over the next few years (Barnes and Yaffe, 2011; Norton et al., 2014; Deckers et al., 2015). The main identified preventable risk factors for AD worldwide are: low education attainment, smoking, physical inactivity, depression, midlife hypertension, diabetes mellitus and mild-life obesity (Norton et al., 2014). Taken together, these seven modifiable risk factors may contribute to 30%–50% of AD cases (Barnes and Yaffe, 2011; Norton et al., 2014).

Since the 1990s, it has been proposed that the brain innate immune response plays an important role in the development and progression of the neurodegeneration in AD (McGeer et al., 1990; McGeer and McGeer, 1995). Such response is characterized by the presence of activated microglia, the resident immune cells of the brain, which increase in cell density and undergoes changes in morphology and in the expression of surface antigens (Perry et al., 2007). Indeed, a number of clinical and experimental studies demonstrated the importance of systemic peripheral inflammation or infection as pivotal contributors to the pathophysiology of AD, supporting a bi-directional communication between brain and peripheral immune systems (Perry et al., 2007; Perry and Teeling, 2013). More recently, it has been proposed that peripheral inflammation/infection may be not just a contributor but indeed a key determinant of the cognitive decline associated to AD progression (Cunningham and Hennessy, 2015).

Interestingly, a considerable worldwide population is affected by periodontal diseases, that are oral infections that affect teeth supporting tissues (Dye, 2012; Eke et al., 2015; Oppermann et al., 2015). They can be classified as gingivitis, when the inflammation is localized in the gingival tissues, or it may assume a more severe destructive form, with the inflammatory process reaching deeper connective and bone tissue, causing bone and attachment loss, that may ultimately lead to tooth loss (Armitage, 1999; Kamer et al., 2008). This local inflammatory process may induce a systemic inflammatory state through mechanisms that include dissemination of pro-inflammatory cytokine and/or bacteria from the oral to extra-oral sites (Hajishengallis, 2015). Therefore, periodontitis could trigger and/or exacerbate an inflammatory condition especially in elderly subjects, leading to memory impairments, contributing to accelerate the progression of neurodegenerative diseases such as AD.

This review will discuss the findings that may clarify the influence of periodontitis on the magnitude of the neuroinflammatory status as well as to highlight experimental and clinical findings indicating a possible comorbidity between periodontitis and AD.

## Role of Neuroinflammation in The Pathogenesis of Alzheimer’s Disease

The pathological hallmarks of AD include the synaptic loss and the presence of senile plaques and neurofibrillary tangles. The senile plaques are primarily composed of β-amyloid (Aβ) peptide, which is a 39–43 amino acidic peptide formed upon proteolytic processing, by β- and γ-secretases (Haass and De Strooper, 1999), of the larger amyloid precursor protein (APP), a ubiquitously expressed transmembrane glycoprotein. The Aβ cascade hypothesis in AD pathogenesis postulates that increased accumulation of Aβ appears to be related to a gradual synaptic loss and neuronal death finally leading to cognitive impairments (Selkoe, 1991; Hsiao et al., 1996; Hardy and Selkoe, 2002).

A contribution of neuroinflammation to the pathogenesis of AD has been pointed since complement factors surrounding senile plaques were observed in post mortem brain tissue from AD patients (Eikelenboom and Stam, 1982; McGeer and McGeer, 2013). Further support to this idea came from a number of epidemiological studies indicating that chronic use of nonsteroidal anti-inflammatory drugs reduces the risk of developing AD (McGeer et al., 1990; in ’t Veld et al., 2001; McGeer and McGeer, 2013), and that several inflammatory mediators are elevated in the brain and cerebrospinal fluid of AD patients (Heneka et al., 2015a). The traditional view postulates that Aβ deposits or oligomers trigger the recruitment and activation of microglia, which release inflammatory mediators that aggravate an already ongoing neurodegenerative process. However, some authors argue that neuroinflammation has a more central role to the pathogenesis of the disease than previously considered (Holmes, 2013; Heneka et al., 2015a,b).

**Figure 1:**
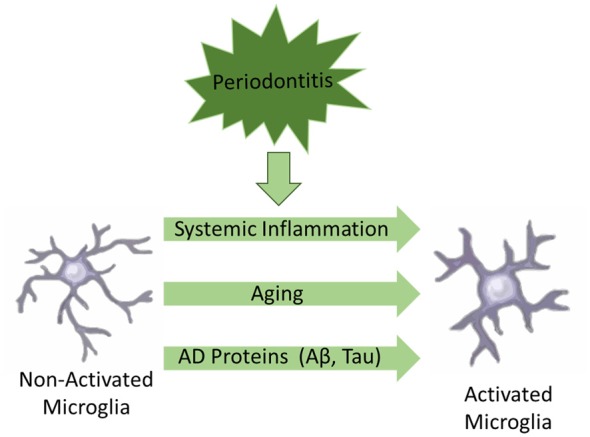
Schematic illustration of factors associated to microglial activation in Alzheimer’s disease (AD).

Another important updated concept is the fact that the central nervous system (CNS) is not an immune-isolated environment, since there is converging evidence of a bidirectional cross-talk between the brain and the peripheral immune system (Holmes, 2013; Perry and Teeling, 2013). The peripheral systemic inflammation may contribute not only to aggravate the progression of neurodegeneration in AD, but could also play a fundamental role in the development of the disease (Holmes, 2013; VanItallie, 2017). The main underlying mechanism is the “priming” of microglia, which postulates that microglia acquire a “primed” phenotype, ready to express a damaging pro-inflammatory response to further insults. Microglia priming could be initially triggered by a number of conditions such as: (i) aging; (ii) proteins associated to AD pathogenesis (e.g., Aβ, tau); and (iii) systemic inflammation (see Figure 1). Further events of systemic inflammation would switch primed microglia to an aggressive pro-inflammatory phenotype contributing to aggravate neuroinflammation and neurodegeneration (Perry and Teeling, 2013). Of note, at least five of the main preventable risk factors for AD, e.g., smoking, depression, hypertension, diabetes mellitus and obesity, have a common association with a systemic pro-inflammatory phenotype, giving further support to the hypothesis that systemic inflammation may play a fundamental role in the development and progression of AD (Holmes, 2013; VanItallie, 2017).

It is important to note that chronic activation of the complement system is also associated with AD (Fischer et al., 1995; Fonseca et al., 2016). Proteins associated to AD can activate complement and recruit activated glia (astrocytes and microglia) to the amyloid plaque, that secrete proinflammatory cytokines or other toxic mediators that could play a role in neuronal degeneration in AD (Fischer et al., 1995; Fonseca et al., 2016).

Preclinical studies have evaluated systemic inflammation by peripheral administration of lipopolysaccharide (LPS) in animals as a model of bacterial infection (Sly et al., 2001; Godbout et al., 2005). It has been observed that peripheral administration of LPS results in increased levels of tumor necrosis factor-α (TNF-α), interleukin-1β (IL-1β) and interleukin-6 (IL-6) in both periphery and the brain (Godbout et al., 2005; Teeling and Perry, 2009), alters the blood-brain barrier (BBB) transport of Aβ protein (Jaeger et al., 2009), increasing the brain levels of Aβ (Sly et al., 2001; Jaeger et al., 2009). This inflammatory response may predispose to neurodegenerative diseases (Godbout et al., 2005) or even amplify the ongoing neurodegenerative process (Sly et al., 2001). In aged laboratory animals, an exacerbated neuroinflammatory response is associated with sickness behavior including depressive-like behavior and cognitive deficits, which are also observed in AD (Godbout et al., 2005). In a prospective longitudinal study, it was observed that increased levels of serum TNF-α following that both acute and chronic systemic inflammation is associated with cognitive decline in AD patients (Holmes et al., 2009).

Some potential crosstalk sites between periphery and the brain are: vagal afferents; structures lacking the BBB such as the circumventricular organs; direct effects on vascular endothelial cells at the BBB; and entry of peripheral immune cells into the brain (Teeling and Perry, 2009; Holmes, 2013). Once in the brain, peripheral inflammatory signaling molecules stimulate microglia, which produce more pro-inflammatory cytokines. In young healthy brains, microglia activation is associated with a reparative inflammatory response, which suppresses the initial pro-inflammatory response. In contrast, in aged and/or diseased brains, where microglia has been already primed, an exacerbated inflammatory response takes place, which accelerates cognitive decline (Holmes, 2013; Cunningham and Hennessy, 2015).

Lastly, the regulatory mechanisms of entry and exit of immune cells from the CNS remain poorly understood (Louveau et al., 2015). The increasing knowledge of the brain lymphatic system may lead to a new point of view of this field in neuroimmunology and bring prospects on the etiology of neuroinflammatory and neurodegenerative diseases related with immune system dysfunctions (Berton et al., 2015). Dysfunctions of the meningeal lymphatic vessels may be the cause of a wide range of neurological disorders, in which altered immunity is a prevalent aspect, such as multiple sclerosis and AD (Akiyama et al., 2000; Berton et al., 2015).

## Contribution of Periodontitis for Neuroinflammation and Diseases

Comorbidity between periodontal disease and AD has been reported in two front lines. The first line of evidence is that AD patients have greater impairment of oral health because of their progressive cognitive impairment, which would affect their oral hygiene habits (Kamer et al., 2008; Mancini et al., 2010; Gaur and Agnihotri, 2015). The second one is that uncontrolled periodontal disease could trigger or exacerbate neuroinflammatory phenomenon observed in AD (Kamer et al., 2008; Teixeira et al., 2014; Gaur and Agnihotri, 2015). However, it must be conceded that interventional studies reporting a direct association between periodontitis and AD are still missing.

**Figure 2:**
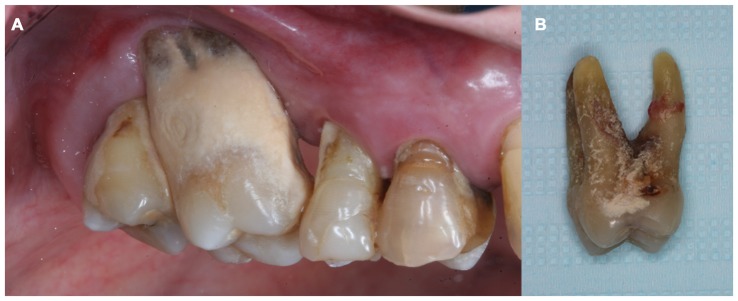
**(A)** Teeth from a chronic periodontal patient presented dental calculus, gingival recession and attachment loss. **(B)** Molar tooth showed in **(A)** extracted due to advanced periodontal disease involvement.

Periodontitis is a chronic inflammatory disease, initiated by gram-negative bacteria that trigger host immuno-inflammatory response leading to tooth apparatus injury (Page and Kornman, 1997; Page, 1998; Watts et al., 2008; Gaur and Agnihotri, 2015). It is clinically characterized by bleeding on probing and clinical attachment loss (CAL; Armitage, 1999). The gums are usually swollen and discolored, dental calculus is frequently found on compromised teeth (Friedewald et al., 2009; Figure 2A), and tooth loss can result if left untreated or after unsatisfactory response to treatment (Page and Kornman, 1997; Figure 2B). Individuals with periodontitis are usually asymptomatic, except when acute processes occur, such as abscess and necrotizing periodontal diseases (Friedewald et al., 2009). Therefore, despite its high prevalence in the adult population of both developed (Eke et al., 2015) and developing countries (Oppermann et al., 2015), periodontitis is usually an unrecognized disease by both patients and health professionals.

In periodontitis, the exacerbated host inflammatory response is associated with greater amount of tissue damage (Page and Kornman, 1997; Kamer et al., 2008). A systemic host immune-inflammatory response against periodontal pathogens is indicated by the presence of antibodies against periodontal pathogens, such as *Porphyromonas gingivalis, Tannerella forsythia* and *Treponema denticola* (Poole et al., 2013; Noble et al., 2014). Additionally, patients diagnosed with periodontitis present higher levels of inflammatory mediators in serum, such as C-reactive protein (CRP) in patients with chronic periodontitis (Bansal et al., 2014; Ardila and Guzmán, 2015), and leptin in patients with aggressive periodontitis (Shi et al., 2015). It has been suggested that the periodontal infection and the immuno-inflammatory response against periodontal pathogens may increase the host susceptibility to systemic diseases, including osteoporosis (Martelli et al., 2017), diabetes mellitus (Hanes and Krishna, 2010; Otomo-Corgel et al., 2012; Preshaw et al., 2012), cancer (Martelli et al., 2017), autoimmunity and cardiovascular disease (Page, 1998; Friedewald et al., 2009; Pejcic et al., 2011; Otomo-Corgel et al., 2012; Martelli et al., 2017), dementia (Pazos et al., 2016) and neurodegenerative diseases such as AD (Kamer et al., 2008, 2015; Rogers, 2008; Kubota et al., 2014; Gaur and Agnihotri, 2015; Ganesh et al., 2017; Sochocka et al., 2017a).

It is worth mentioning that a wrong clinical diagnosis of AD can generate other confounding factors. Vascular dementia, a subtype of dementia, such as AD (Appleton et al., 2017), may also be modulated indirectly by periodontitis, since periodontitis may be associated with clinical signs of atherosclerosis (Nakib et al., 2004; Yang et al., 2013; Etemadifar et al., 2015; Ahn et al., 2016), and atherosclerosis contribute to the development of vascular dementia (Appleton et al., 2017).

## Periodontitis and Alzheimer’s Disease

This possible comorbity between periodontitis and AD has been indicated by clinical studies comparing the presence of periodontitis in individuals with and without AD (Stein et al., 2012; Martande et al., 2014; Noble et al., 2014; Cestari et al., 2016). One of these studies described an association between inflammatory cytokine levels in patients with AD and periodontitis, suggesting that periodontitis may be associated with onset, progression and aggravation of AD (Cestari et al., 2016). Similar results were described for serum IgG antibody levels to bacteria associated with periodontitis, observing an increasing incident AD onset/progression among participants with high serum antibody (Stein et al., 2012; Noble et al., 2014). When comparing periodontal health status, individuals with AD present a worsening of the condition with the progression of periodontitis, in which the probing depth and clinical attachment level, clinical parameters for periodontitis, were much higher in AD groups when compared to individuals without AD (Martande et al., 2014).

Periodontal pathogens and the immuno-inflammatory host response in periodontitis may affect the brain function, especially in more vulnerable elderly subjects, and may contribute to onset and progression of neurodegenerative disorders (Kamer et al., 2009). Some putative mechanisms that could explain how periodontitis may affect the homeostasis of the CNS have described by experimental studies and include: (i) translocation of bacteria into blood stream (bacteremia) or invasion into the brain via trigeminal nerve (e.g., *Porphyromonas gingivalis*); and (ii) production of pro-inflammatory cytokines that enter into the blood stream and act systemically or that reach the brain via peripheral nerves pathway (Gurav, 2014; Abbayya et al., 2015; Cerajewska et al., 2015; Gaur and Agnihotri, 2015; Olsen et al., 2016; Ganesh et al., 2017; Nezu et al., 2017; Sochocka et al., 2017a,b).

The first hypothesis relies on the fact that the microorganisms located on the dental biofilm can infiltrate in the brain by blood stream or peripheral nerves, mainly by trigeminal nerves (Riviere et al., 2002). Approximately 85% of the subgingival biofilm is composed by LPS-containing Gram-negative bacteria (Socransky and Haffajee, 2002). Such microorganisms and their immunogenic compounds in certain concentrations can trigger an inflammatory process in the CNS (Abbayya et al., 2015). This inflammatory process is a classic immune response similar in some aspects to that observed in AD, through TLR-2 and TLR-4 pathway, also related to cytokines interactions (including interleukins, TNF-α, transforming growth factor-β) and chemokines (monocyte chemotactic protein, IL-8, macrophage migration inhibitory factor and monokine induced by γ-interferon) released by neurons and glial cells. In addition, it is also observed increased production of reactive oxygen species (ROS) and reactive nitrogen species (RNS) as well as complement system activation. The association of these factors triggers mechanisms of cell death and increases chronic inflammation already established by resident diseases or contribute to the development of new pathologies (Akiyama et al., 2000; Laflamme and Rivest, 2001; Gasque, 2004; Olson and Miller, 2004; Qin et al., 2005; Weller et al., 2009). Such hypothesis is supported by findings from independent research groups showing that peripheral infections can cross over the CNS (MacIntyre et al., 2003; Miklossy et al., 2006; Hammond et al., 2010). Of high interest, LPS has been linked to increased neuronal Aβ peptides levels and consequent disruption of BBB permeability and brain damage in animal models of AD (Lee et al., 2008; Jaeger et al., 2009).

Besides the inflammatory process generated by bacteria infection, some authors claim that there are subjects with “inflammatory traits” inherited which are similar to a vulnerability of the development of the neuroinflammatory diseases and the bacteria infection on the CNS may trigger the exaggerated innate immune response (van Exel et al., 2009; Singhrao et al., 2015; Olsen et al., 2016). Interestingly, periodontitis has been described to increase the gravity of AD in Down syndrome (DS) subjects (Kamer et al., 2016).

**Figure 3:**
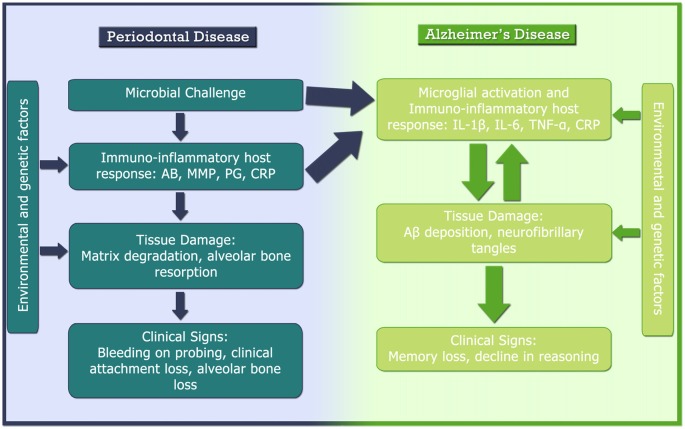
Pathogenesis of AD and periodontal disease and their relationship. AB, antibody; Aβ, β amyloid protein; BoP, bleeding on probing; CAL, clinical attachment loss; CRP, C-reactive protein; IL, interleukin; MMP, matrix metalloproteinase; PG, prostaglandin; TNF-α, tumor necrosis factor-α.

Although the access of periodontal bacteria into the neural parenchyma may be a factor which contributes to the aggravation and acceleration of the AD progression, the neuroinflammatory mechanisms from a systemic inflammatory response triggered by periodontitis has been more consistent in the literature. As illustrated in Figure 3, several studies have shown a positive relationship between CRP blood levels and periodontitis (Ebersole et al., 1997; Fredriksson et al., 1999; Loos et al., 2000; Noack et al., 2001; Slade et al., 2003). CRP is a plasma protein that participate in the systemic response to inflammation and it is regulated by cytokines like IL-6, IL-1β and TNF-α, that trigger the production by hepatocytes (Craig et al., 2003), in which have been reported as a sensible marker of systemic inflammation (Barrientos et al., 2010). Additionally, increased levels of TNF-α in the systemic circulation of AD patients has been related to the presence of periodontopathogenic microorganisms (i.e., *Aggregatibacter actinomycetemcomitans, Tannerella forsythia* and *Porphyromonas gingivalis*) as well as the antibodies against such pathogens (Kamer et al., 2009; Olsen et al., 2016).

Among the pathogens related to periodontitis, the *P. gengivalis* has been related to the levels of CRP found in the aged patients (Winning et al., 2015). Such relationship among this pathogen and the high levels of CRP clarify the positive correlation already described in a study, in which the patients with severe periodontitis have increased serum levels of CRP when compared with unaffected control population (Gomes-Filho et al., 2011). Besides, the level of CRP increases subsequently with the severity of the periodontal disease (Bansal et al., 2014).

In fact, a recent study showed a positive association between periodontal disease and brain Aβ load in humans (Kamer et al., 2015). These findings are consistent with previous animal studies data showing that peripheral inflammation/infections are sufficient to induce brain Aβ accumulation (Kamer et al., 2015). Moreover, the periodontal health status of individuals with AD deteriorates with disease progression and is closely related to their cognitive function (Martande et al., 2014; Sochocka et al., 2017b) and emotional disorders (Kiecolt-Glaser et al., 2002). Corroborating these findings, another study revealed a significant increase in the serum levels of TNF-α in patients with AD and chronic periodontitis in comparison to patients with AD and healthy periodontium (Farhad et al., 2014).

Abe et al. (2011) described increased levels of Aβ precursor protein (APP) in patients with chronic periodontitis. APP is recognized to play an important role in the pathophysiology of AD, increasing the accumulation of Aβ in the CNS (Otsuka et al., 1991) and can directly be modulated by NF-κB (Chami et al., 2015) and TNF-α (Keller et al., 2013), that are also elevated in periodontal diseases.

Considering the increasing recognition of periodontitis as an environmental modifiable factor for AD, recently it has been proposed that the adequate treatment or prevention of periodontal disease may represent a valuable strategy to prevent (or delay) AD development as well as to counteract AD progression (Kamer et al., 2016). However, it is also important to consider that mild systemic inflammatory response, as caused by periodontitis, before to an injury to CNS, may exert a neuroprotective effect in a rat model of ischemic stroke, minimizing the inflammatory response which usually occurs in response to stroke (Petcu et al., 2008).

## Final Considerations

The findings reviewed here clearly pointed inflammation as an important role in both periodontitis and AD. Since periodontitis is a preventable and treatable factor, subjects diagnosed with periodontitis should be informed and treated in an effort to diminish the microbial challenge and the pro-inflammatory cytokines hyper-production, aiming to promote a better quality of life, especially in elderly period. More importantly, despite the existence of clinical studies indicating the comorbidity of periodontitis and AD and the identification of serum antibodies to periodontal pathogens in AD, there is no study showing clearly the causal link between periodontitis and AD.

## Author Contributions

All authors wrote the manuscript. MTS graphed the illustration. All authors discussed and edited the manuscript. All authors read and approved the final manuscript.

## Conflict of Interest Statement

The authors declare that the research was conducted in the absence of any commercial or financial relationships that could be construed as a potential conflict of interest.
